# Hot Weather and Suicide Deaths among Older Adults in Hong Kong, 1976–2014: A Retrospective Study

**DOI:** 10.3390/ijerph17103449

**Published:** 2020-05-15

**Authors:** Pui Hing Chau, Paul Siu Fai Yip, Eric Ho Yin Lau, Yee Ting Ip, Frances Yik Wa Law, Rainbow Tin Hung Ho, Angela Yee Man Leung, Janet Yuen Ha Wong, Jean Woo

**Affiliations:** 1 School of Nursing, The University of Hong Kong, Hong Kong, China; janetyh@hku.hk; 2 The Hong Kong Jockey Club Centre for Suicide Research and Prevention, The University of Hong Kong, Hong Kong, China; sfpyip@hku.hk (P.S.F.Y.); flawhk@hku.hk (F.Y.W.L.); 3 Department of Social Work and Social Administration, The University of Hong Kong, Hong Kong, China; tinho@hku.hk; 4 School of Public Health, The University of Hong Kong, Hong Kong, China; ehylau@hku.hk; 5 The Duchess of Kent Children’s Hospital at Sandy Bay, Hong Kong, China; iyt003@ha.org.hk; 6 Centre of Bebavioral Health, The University of Hong Kong, Hong Kong, China; 7 School of Nursing, The Hong Kong Polytechnic University, Hong Kong, China; angleung@hku.hk; 8 Department of Medicine & Therapeutics, The Chinese University of Hong Kong, Hong Kong, China; jeanwoowong@cuhk.edu.hk

**Keywords:** Suicide, temperature, weather, older adults, Hong Kong

## Abstract

Findings of the association between hot weather and suicide in a subtropical city such as Hong Kong are inconsistent. This study aimed to revisit the association by identifying meteorological risk factors for older-adult suicides in Hong Kong using a time-series approach. A retrospective study was conducted on older-adult (aged ≥65) suicide deaths in Hong Kong from 1976 to 2014. Suicides were classified into those involving violent methods and those involving nonviolent methods. Meteorological data, including ambient temperature, were retrieved. Transfer function time-series models were fitted. In total, 7314 older-adult suicide deaths involving violent methods and 630 involving nonviolent methods were recorded. For violent-method suicides, a monthly average daily minimum ambient temperature was determined to best predict the monthly rate, and a daily maximum ambient temperature of 30.3 °C was considered the threshold. For suicide deaths involving nonviolent methods, the number of days in a month for which the daily maximum ambient temperature exceeded 32.7 °C could best predict the monthly rate. Higher ambient temperature was associated with more older-adult suicide deaths, both from violent and nonviolent methods. Weather-focused preventive measures for older-adult suicides are necessary, such as the provision of more public air-conditioned areas where older adults can shelter from extreme hot weather.

## 1. Introduction

Suicide is a global public health problem. Well-known risk factors of suicide among older adults include socio-demographic characteristics (e.g., older age, financial debt), social factors (e.g., social support, social relationships, stressful life events), and clinical factors (e.g., physical disability, physical illnesses, pain, depression, psychiatric illness, cognitive impairment) [1,2,3]. Apart from these factors, hot weather is also reported to be a risk factor of suicide [4,5,6]. 

The rate of suicides generally increases with ambient temperature [7]. This association was reported in East Asia [8,9], Europe [10,11,12,13], America [14], and in the Southern Hemisphere [15,16]. Positive associations between suicide rates and sunshine duration [10,17,18], and temperature fluctuations [19,20,21,22] were also reported. Furthermore, the extent to which suicides are affected by climatic factors is related to the methods used in suicide attempts. Studies have reported that suicides by nonviolent methods (e.g., poisoning) were less likely to be associated with hot weather, compared to suicides resulting from violent methods (e.g., jumping from a height, hanging, cutting oneself) [7,23,24]. Moreover, the older population is more vulnerable to heat stress because of weakening thermoregulations with age [25].

Hong Kong has one of the highest suicide mortality rates worldwide for individuals aged 65 or older, with a rate in 2014 of 25 per 100,000 people [26]. The majority of older-adult suicide deaths in Hong Kong involved violent methods such as jumping from a height (56.3%) and hanging (28.3%) [26]. 

Hong Kong has a subtropical climate. Its summer begins in May and ends in September. Between 1981 to 2010, the monthly average daily maximum ambient temperature was approximately 30 °C and the monthly average daily minimum ambient temperature was approximately 26 °C. The highest daily maximum ambient temperature recorded was 36.6 °C, on August 22, 2017, and instances of extreme heat and rainfall are becoming more frequent. In winter, the monthly average daily maximum ambient temperature drops to approximately 20 °C and the monthly average daily minimum ambient temperature to 15 °C. Studies have reported that deaths from circulatory and respiratory diseases in Hong Kong are more closely related to cold than hot weather [27,28,29,30]. However, data regarding the impact of meteorological variables as risk factors for suicide deaths or attempts in Hong Kong remain inconsistent [31,32,33].

The development of appropriate suicide interventions, particularly for the older population in Hong Kong, requires an examination of the association between suicide deaths and meteorological variables. This would also contribute to similar investigations regarding other cities with subtropical climates. This study used a time-series approach, focusing on nearly four decades of data, adjusted for various meteorological variables and accounting for time-related confounding factors (such as festivals), in order to address the existing inconsistencies in the previous findings from a new perspective. Specifically, the objective of this study was to identify meteorological-based risk factors related to older-adult suicide deaths in a city with a subtropical climate, thereby addressing the existing inconsistencies in current related research. 

## 2. Materials and Methods

### 2.1. Data

A retrospective study was conducted. All suicide deaths among the Hong Kong population involving individuals aged 65 years or above and that occurred between 1976 and 2014 were requested from the Census and Statistics Department of Hong Kong. Causes of death were classified using the International Classification of Diseases (ICD). Table 1 shows the ICD codes for identifying suicides, separated into violent and nonviolent methods. Deaths with an unknown cause, month, or year of death were excluded. Population statistics were also obtained from the Census and Statistics Department. The population statistics were released for mid-year and end-year. Hence, mid-year estimates were used for April to September and end-year estimates were used for October to March the next year.

Statistics regarding daily meteorological variables, namely, mean ambient temperature, daily maximum ambient temperature, daily minimum ambient temperature, sunshine duration, cloud amount, dewpoint, and typhoon signals were obtained from Hong Kong Observatory. These variables were chosen in light of the common factors studied in association with suicide [7]. Since evidence exists regarding a possible association between suicides and festivals [34,35], we also extracted information concerning the dates of various festivals. 

As living human subjects were not involved, ethics approval was exempted. Data was subject to third party restrictions.

### 2.2. Data Analyses

Suicide deaths were aggregated into monthly data and adjusted for the number of days in each month. The monthly suicide mortality rate was calculated by dividing the adjusted number of suicide deaths by the corresponding population size. Deaths registered before 1995 did not have a death date available, and even for deaths registered after 1995, one third of them had only the month of death but not the day of death. Suicide deaths from violent methods and nonviolent methods were analyzed separately. The daily meteorological data were transformed into 15 variables that could be used for the data analysis (Appendix Table A1).

After applying specific threshold values (28 °C and 33 °C for ambient temperature) to define extreme weather, sensitivity analysis based on various percentiles of the meteorological variables was conducted to determine the optimal threshold values for extreme weather. For the 75th, 95th, and 99th percentiles, the adjusted monthly number of days with a meteorological variable with a value larger than those percentiles was counted. For the first, fifth, and 25th percentiles, the adjusted monthly number of days with a meteorological variable with a value that was less than those percentiles was counted; this also helped to explore if extreme cold weather is associated with more suicides. The percentiles used for the daily minimum ambient temperature were 8.9 °C, 12.1 °C, 17.2 °C, 25.7 °C, 27.9 °C, and 28.6 °C; and the percentiles used for the daily maximum ambient temperature were 13.3 °C, 16.3 °C, 21.4 °C, 30.3 °C, 32.7 °C, and 33.7 °C. 

The total number of festivals in each month was counted. Two series of festivals were created. First, major Chinese festivals, comprising Chinese New Year, the Dragon Boat Festival, the Mid-Autumn Festival, and the Winter Solstice Festival, were combined, as these are the most significant festivals for Chinese people. Second, celebratory festivals, comprising the major Chinese festivals and the Western New Year, Chinese Valentine’s Day, Valentine’s Day, Easter, Mother’s Day, Father’s Day, and Christmas, were combined, as these are festivals that are commonly celebrated in Hong Kong. Unlike Western festivals, Chinese festivals are defined according to the lunar calendar. For example, Chinese New Year sometimes occurs in January and sometimes in February. Therefore, the festivals do not occur in the same month.

We adopted a time-series approach which could account for the autocorrelative nature of the data points [36]. First, trend and seasonality were assessed by fitting Autoregressive Integrated Moving Average (ARIMA) models. Then, transfer function models that involved the inclusion of meteorological variables as explanatory variables were fitted to identify risk factors of older-adult suicide deaths. The transfer function models were fitted one-by-one for each of the meteorological variables. Model identification was based on the iterative approach applied by Box and Jenkins [36]. The residual autocorrelation and partial autocorrelation functions were examined. Ljung-Box test was used to examine residuals after fitting the time series model, with the null hypothesis that autocorrelations up to lag k equal zero (i.e., white noise). Goodness-of-fit was shown by an insignificant Ljung-Box test. There was no gold standard in choosing the lag (k) in the Ljung-Box test. We used lag 18 in this study because it was used in the literature. Therefore, a p-value for the Ljung-Box test (lag 18) of greater than 0.05 and p-values for the fitted coefficients of less than 0.05 were considered acceptable. The meteorological variable that had the lowest Schwarz’s Bayesian Information Criterion (SBC) value was selected and considered to be the best predictor of the suicide mortality rate. Then, a second explanatory series was added to the model to examine if there was any improvement in terms of model fit. Here, meteorological variables and the two series of variables reflecting the monthly number of Chinese festivals and celebratory festivals were inserted individually. Finally, sensitivity analysis using other thresholds was attempted to examine if a better fit could be achieved. 

Mean Absolute Scaled Error (MASE) was used to quantify the accuracy of the models [37]. A MASE below 1 implied that the fitted model gave smaller errors than the errors from the naïve method. A MASE far greater than 1 implied poor accuracy. A seasonal naïve forecast was adopted as there was seasonality in the time series.

SAS version 9.4M4 was used for statistical analyses. 

## 3. Results

### 3.1. Overview

**Figure 1:**
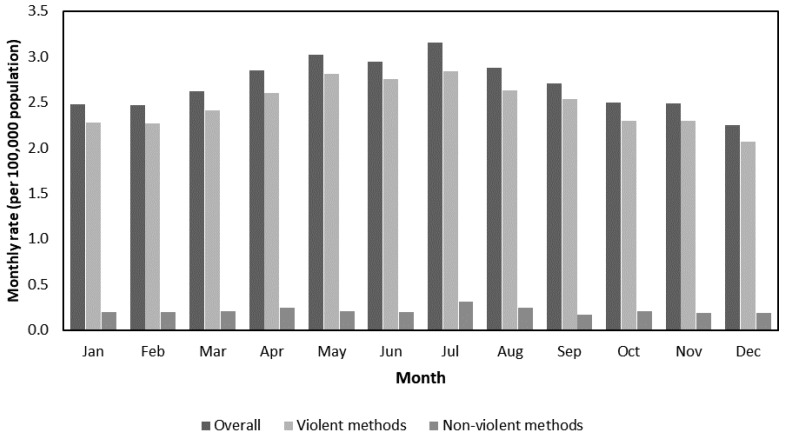
Average monthly rates^#^ of older-adult suicide deaths in Hong Kong, 1976–2014. ^#^ Adjusted to 30 days in a month.

During 1976 to 2014, there were 7,314 older-adult suicide deaths from violent methods and 630 older-adult suicide deaths from nonviolent methods in Hong Kong. In 2014, the annual older-adult suicide mortality rate from violent methods was 22.7 per 100,000 population, and that from nonviolent methods was 2.4 per 100,000 population (Appendix Table A2). The average monthly rates of older-adult suicide deaths—both by violent and nonviolent methods—peaked at July (Figure 1). For the monthly rates of older-adult suicide mortality from violent methods, both first-order differencing and seasonal differencing were applied in the best-fitting ARIMA model: ARIMA(1,1,2) × (0,1,1)_12_. Diagnostics statistics indicated an acceptable fit, with SBC equaling −2957.8 and the p-value of the Ljung-Box test (lag 18) being 0.321. Meanwhile, for the monthly rates of older-adult suicide mortality from nonviolent methods, first-order differencing was applied in the best-fitting ARIMA model, ARIMA (0,1,1), without seasonality. Diagnostics statistics indicated an acceptable fit, with SBC being −3993.6 and the p-value of the Ljung-Box test (lag 18) being 0.084. 

Regarding the meteorological variables related to hot weather, all had seasonal differencing applied in the best-fitting ARIMA models, and this implied that the monthly statistics had a stable trend when compared to the same month from the preceding year. Descriptive statistics indicated that the trends were increasing (Appendix Table A2). All considered meteorological variables demonstrated a clear annual seasonal pattern. Table 2 shows the best-fitting ARIMA models for the meteorological series.

### 3.2. Meteorological Risk Factors of Older-Adult Suicides 

#### 3.2.1. Violent Methods

**Figure 2:**
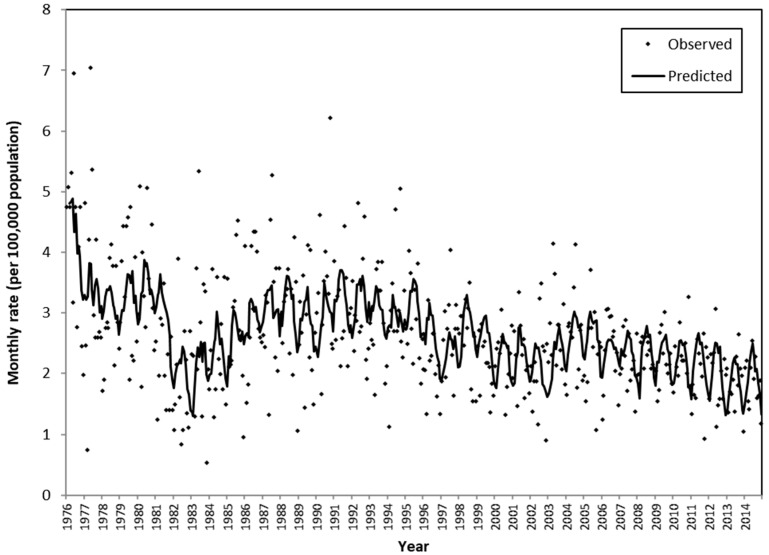
The observed rate of older-adult suicide mortality from violent methods and the predicted rate based on the transfer function model that featured monthly average daily minimum ambient temperature as the input series.

For all of the models for suicide deaths through violent methods, first-order differencing was applied and the MA(3) model was used for the noise series. It was found that ambient temperature, ambient temperature change within a week, dewpoint, amount of cloud, sunshine hours, total rainfall, ambient temperature above a threshold, and typhoons were associated with older-adult suicide mortality through violent methods (Table 3). The model with monthly average daily minimum ambient temperature had the lowest SBC value (−3078.71). Further, the sensitivity analysis showed that the input series created by the various percentiles did not attain a better fit than the model featuring monthly average daily minimum ambient temperature (Appendix Table A3). Furthermore, if another meteorological input series, celebratory festival, or Chinese festival was added to the model, the model fit did not improve. Hence, monthly average daily minimum ambient temperature was determined to be most useful for predicting older-adult suicide deaths from violent methods. (Figure 2) The transfer function model was as follows:

(1) $$\left(1-B\right)Y_{t}=\frac{\left(0.0005-0.0006B\right)}{\left(1-0.86B\right)}\left(1-B\right)\left(X_{t}\right)+\left(1-1.00B-0.05\;B^{2}+0.16\;B^{3}\right)a_{t}$$

where $Y_{t}$ denotes older-adult violent suicide mortality rate at month t, $X_{t}$ denotes the monthly average daily minimum ambient temperature at month t, and $a_{t}$ denotes Gaussian white noise. B is the backshift operator, such that $BX_{t}=X_{t-1}$. All the fitted coefficients had a *p*-value < 0.001, except the coefficient for *B*^2^ had a *p*-value of 0.443. A MASE of 0.77 was obtained. Cross correlation between the two time series at lag 0 was 0.11. The model implies that, among the older population, higher monthly mean daily minimum temperature is associated with a higher monthly rate of suicide deaths through violent methods. 

Interestingly, some meteorological input series constructed based on the 25th percentile had comparable fit to the above model. Specifically, more days within a month with a daily maximum ambient temperature below 21.4 °C (SBC = −3064.9), and more days within a month with a daily minimum ambient temperature below 17.2 °C (SBC = −3062.01) were associated with lower monthly suicide deaths from violent methods, implying a possible protective effect of cool weather. On the other hand, among all meteorological input series constructed based on the 75th, 95th, and 99th percentiles, the model with a monthly number of days with daily maximum ambient temperature exceeding 30.3 °C gave the best fit. In other words, if a threshold is necessary, 30.3 °C could be suitable.

#### 3.2.2. Nonviolent Methods

**Figure 3:**
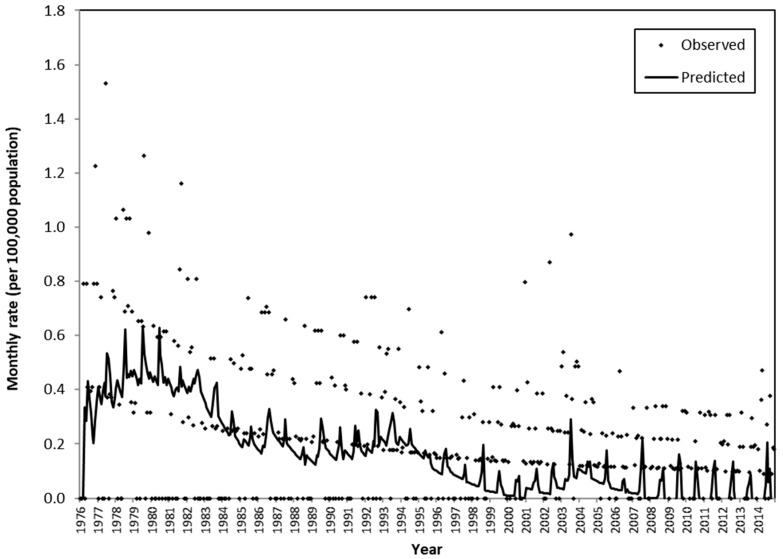
The observed rate of older-adult suicide mortality from nonviolent methods and the predicted rate based on the transfer function model that featured an adjusted monthly number of days with daily maximum ambient temperature exceeding 32.7 °C as the input series.

For all of the models concerning suicide mortality through nonviolent methods, first-order differencing was applied and the MA(1) model was used for the noise series. It was found that ambient temperature, ambient temperature range within a week, dewpoint, amount of cloud, sunshine hours, total rainfall, ambient temperature above a threshold, and typhoons were associated with older-adult suicide deaths through nonviolent methods (Table 4). The model with monthly average daily maximum ambient temperature had the least SBC (−3993.5). The sensitivity analysis using an adjusted number of days with a daily maximum ambient temperature exceeding 32.7 °C (95th percentile) within a month as its input series showed a smaller SBC (−3994.4) than that with monthly average daily maximum ambient temperature as its input series, while maintaining a *p*-value of 0.087 for the Ljung-Box test (lag 18) (Appendix Table A4). Therefore, this model was considered as the best model for the mortality rate of older-adult suicides from nonviolent methods. If another meteorological input series, celebratory festivals, or Chinese festivals were added to the model, it did not improve the model fit. Hence, the number of days in a month with daily maximum ambient temperature exceeding 32.7 °C was determined to be most useful for predicting older-adult suicide deaths from nonviolent methods (Figure 3). The transfer function model was as follows:

(2) $$\left(1-B\right)Y_{t}=\left(0.00012\right)\left(1-B\right)\left(X_{t}\right)+\left(1-0.96B\right)a_{t}$$

where $Y_{t}$ denotes older-adult nonviolent suicide mortality rate at month t, $X_{t}$ denotes the monthly average number of days with daily maximum ambient temperature exceeding 32.7 °C at month t, and $a_{t}$ denotes Gaussian white noise. B is the backshift operator, such that $BX_{t}=X_{t-1}$. All the fitted coefficients had a *p*-value < 0.001. A MASE of 1.00 was obtained. Cross correlation between the two time series at lag 0 was 0.04. The model showed that the more days in a month for which the maximum daily temperature exceeds 32.7 °C, the higher the rate of monthly suicides from nonviolent methods. This implies that 32.7 °C can be considered a threshold.

## 4. Discussion

This was the first study to demonstrate the association between hot weather and older-adult suicide mortality rates in a subtropical city (Hong Kong) using a time-series approach. For suicide by violent methods, the older-adult mortality rate was found to increase with escalations in monthly average daily minimum ambient temperature, and 30.3 °C could be considered a threshold for daily maximum ambient temperature. For suicide by nonviolent methods, the older-adult mortality rate was found to increase with the monthly number of days having a daily maximum ambient temperature exceeding 32.7 °C. These findings could be generalized across regions with a subtropical climate. Moreover, the methodology could be applied to regions with other climates. 

In the present study, the rate of violent suicide deaths among older adults demonstrated clear seasonality, but nonviolent suicide deaths did not, despite both types of suicide being associated with meteorological variables based on the findings. This result is consistent with reports from previous studies regarding the diminishing of seasonality on suicide deaths [38,39]. Controlling for seasonality in transfer function would reveal the association with seasonal temperature anomalies, whereas not controlling for seasonality would show the seasonal maximum. This study adopted the latter approach. In future research, the former approach should also be attempted.

Our findings were also consistent with existing reports that hot weather is associated with more older-adult suicides involving violent methods [7,8,9]. While some studies have suggested a lack of association between suicide involving nonviolent methods and ambient temperature [24,32], the present study found an association. This finding suggested that suicide, whether by violent or nonviolent methods, was related to hot weather. 

The mechanism of how hot weather affects intention to complete a suicidal act remains uncertain. Researchers have commented that the associations between hot weather and violent and nonviolent methods could be related to the accessibility of methods [40]. However, in Hong Kong, the dominant suicide methods (i.e., jumping from a height, and poisoning by pills or by carbon monoxide generated from charcoal burning) are available throughout the year. Other researchers have suggested that sunshine affects the impact of serotonergic medication [21], or that the diurnal temperature range causes hyperactivity in brown adipose tissue [20]. However, the research results found that ambient temperature is a better predictor of suicide deaths than either sunshine hours or diurnal temperature. Older people have reduced Hypothalamic-Pituitary-Adrenal (HPA) axis activity, which is associated with some forms of depression and suicidal behavior [41,42,43]. Therefore, changes in cortisol levels and HPA-axis reactivity in response to ambient temperature is a possible explanation. Although an increasing but insignificant trend of urban heat island intensity in Hong Kong over the years 1989−2015 has been reported, a significant increasing trend of intensity of extreme urban heat island events in summer has also been reported [44]. The urban heat island effect has been shown to worsen health conditions [45]; thus, Hong Kong’s congested living environment and lack of ventilating design may also exacerbate the suicide risk during extreme hot weather. More studies are needed to investigate this mechanism.

It was noted that despite the increasing trend in ambient temperature, suicide death rates experienced a decreasing trend over the study period. Although there was a positive association between hot weather and suicide rates, this association could only explain part of the variations in suicide rates. The increase in suicides owing to heat stress may be compensated by year-round suicide prevention strategies implemented over the study period. 

According to the Hong Kong Observatory, the mean temperature in Hong Kong has risen by 0.21 °C per decade and the number of days with extreme hot weather is increasing [46]. In 2017, there were 120 days with daily maximum ambient temperatures exceeding 30.3 °C, and 43 of them exceeded 32.7 °C. Over 80% of the days from June to September 2017 and one third of the days from May to October 2017 had daily maximum ambient temperatures exceeding 30.3 °C. This increase in the number of days with extremely hot weather may lead to a consequent increase in older-adult suicide acts in the future. Furthermore, the earliest very hot day was recorded in May and the last very hot day was recorded in October. This implies that extremely hot weather not only occurs in Hong Kong during the usual summer months (June to August), but also that it can occur over a six-month period (from May to October). Therefore, in addition to implementing suicide-prevention measures in summer months, preventive measures should be implemented whenever the daily maximum ambient temperature reaches 30.3 °C in other months. Since a nine-day weather forecast of maximum and minimum ambient temperatures is available from the Hong Kong Observatory, weather-driven preventive measures are feasible. Protocols of the preventive measures must be developed prior to the hot season. Whenever there is a forecast of extremely hot weather, the weather-driven interventions, such as the provision of more public air-conditioned areas where older adults can shelter from extreme hot weather, can be executed. However, there currently appears to be a lack of evidence-based practices regarding weather-driven interventions for suicide prevention, both locally and globally. 

To prevent suicides during periods of hot weather, efforts at different levels need to be pooled, from policymakers, to health and social service providers, as well as the lay public. In the short term, additional phone calls or outreach shall be made to older people by nongovernmental organizations. At present, extra phone calls or outreach services are available in winter. However, these extra services should also be provided during hot days in summer, particularly days with a maximum ambient temperature of 30.3 °C or above, in order to proactively contact service clients with whom service providers have infrequent contact in order to assess their emotional wellness and provide appropriate counselling. As cool conditions may have a protective effect, older people should be encouraged to seek well-ventilated or air-conditioned shelters. At present, common areas of 20 community halls or community centers in Hong Kong will be opened during the daytime on very hot days. However, services are not available during public holidays or Sundays. Moreover, in order to attract older people to utilize these heat shelters, activities will need to be organized for the users. For example, elderly community centers can be opened as heat shelters and offer activities that promote positive thinking and enhance mental wellness. Although 19 temporary night heat shelters will be opened for citizens to stay overnight during hot nights, these are not attractive to older people because they would be staying away from their home and would need to pack their belongings every morning. In the medium term, the government may consider providing an allowance as a form of standardized measure for public housing to install air conditioners, as well as means-tested electricity subsidies to older people in order to support those who cannot afford air conditioning at their home due to financial strains. Indeed, there are older adults who live in suboptimal housing such as subdivided flats; the government may arrange for proper housing, similar to public housing, for them. In the long run, better ventilated housing designs should be adopted for housing development and urban planning will need to take into account the health effects of heat. Nevertheless, despite the provision of a cool environment, older adults are still subject to the heat stress, particularly when they are outdoors. Better urban planning shall also be adopted to minimize heat island effect. Jumping is the most common method of suicide in Hong Kong, where a roof or open staircases are hotspots for jumping. Before the actual jump, most of those considering suicide wander around the hot spot. Security guards of residential blocks should inspect rooftops and open staircases more frequently in order to check for older people who may be considering a suicide act. If security guards can seize this moment, tragedy can be avoided. It may happen that the person might find another way to attempt suicide, but before he/she can find a substitute, that period could be sufficient for social workers or other professionals to step in and provide emotional support. Due to family downsizing, older people living alone is not uncommon. Family caregivers, and the public in general, should pay more attention to signs of depression or suicidal thoughts among older people, particularly those living alone, and if necessary refer such individuals to professional services for support. Among these recommendations, some would be implemented for the entire summer, such as providing subsidies or providing mental wellness programs, while some would be planned ahead and executed on days with extreme hot weather, such as the frequent inspection of potential jumping hotspots and outreach (via phone call or visits) to older adults. It should be remembered that in parallel with weather-driven suicide prevention strategies, interventions targeting at-risk factors at the individual level should be implemented [47]. Furthermore, most of these preventive measures—such as extra phone calls and outreach services, mental wellness programs, heat shelters with meaningful activities, fuel subsidies, proper housing, and urban design—are also applicable to other places. Nevertheless, slight modifications may be needed to accommodate the local features. For example, when providing heat shelters, accessibility should also be considered for more remote neighborhoods; frequent inspection of suicide hotspots should take into account the most common methods or locations of suicide rather than confining checks to residential buildings.

The strength of this study is that it utilized a vast variety of meteorological variables in its investigation and took contextual factors, like festivals, into consideration. An attempt was made to identify a threshold to facilitate the triggering of public health messages. The use of a time-series approach allowed the present study to examine the autocorrelative nature of the data points, but the interpretations of models might not be straightforward. After we put forward the consistent results with models which did not take into account autocorrelative nature, future studies can revert to the simpler models for more straightforward interpretations. 

The study had some additional limitations. First, by using population-level data, we could not consider individual risk factors. While it has been reported that psychiatric illness was the most prominent risk factor in older-adult suicides, [1] our study could not reveal how the risk was contributed to by environmental factors compared with individual factors. Future studies shall include both environmental factors and individual factors in the same study. Although information of gender was available, owing to the small number of suicide cases, particularly for suicides by nonviolent means, the substantial proportion of zero values would make the model estimation perform poorly. For suicide by violent methods, the problem is milder than that for the nonviolent methods. However, we used the same breakdown for both methods for comparison purposes. Hence, analyses were not conducted for males and females separately. The limitation of the small number of nonviolent suicides in Hong Kong has already been noted. It would be worthwhile to conduct similar analyses in other countries or regions with large numbers of nonviolent suicides in order to confirm the observed findings. Moreover, the dates of the suicide acts were unavailable. Consequently, it is possible that for some deaths there was a delay between the suicide act and the date of the death. Furthermore, analysis based on daily statistics is preferred. As the day of death was not available in these records, only monthly data could be utilized, which might not be the most appropriate for analyzing effects and lagged effects caused by daily fluctuations in weather or by the holding of festivals. This study only attempted to examine a limited number of meteorological variables and used a limited definition of an extended period (three consecutive days) of extreme weather as predictors. In the future, daily analysis will be attempted. Furthermore, other metrics for weather such as a composite heat index, as well as various definitions of prolonged periods of extreme temperature, could be used as predictors in the analysis. Moreover, future studies should investigate whether heat stress also applies to the younger population and to the entire population regardless of age.

## 5. Conclusions

Higher ambient temperature was associated with more older-adult suicide deaths, both from violent and nonviolent methods. Weather-focused preventive measures for older-adult suicides are necessary, such as the provision of more public air-conditioned areas where older adults can shelter from extreme hot weather.

## Figures and Tables

**Table 1:** International Classification of Diseases (ICD) used for identification of suicide deaths.

Year	ICD-Version	Suicide by Violent Methods	Suicide by Nonviolent Methods
1976−1978	ICD-8	1953–1957	1950–1952
1979−2000	ICD-9	E953–E957	E950–E952
2001−2009	ICD-10	X70–X82	X60–X69
2010−2014	ICD-10 (updated)	X70–X82	X60–X69

Note: Violent methods include hanging, drowning, using firearms on oneself, cutting oneself, and jumping from a height. Nonviolent methods include poisoning.

**Table 2:** ARIMA models for the meteorological variables related to hot weather.

Meteorological Variable	Best ARIMA Model	SBC	P-Value of Ljung-Box Test (Lag 18)
Monthly average of daily maximum ambient temperature	(0,0,1) × (0,1,1)_12_	1348.4	0.564
Monthly average of daily mean ambient temperature	(0,0,1) × (0,1,1)_12_	1280.9	0.492
Monthly average of daily minimum ambient temperature	(0,0,1) × (0,1,1)_12_	1325.6	0.297
Monthly average of diurnal ambient temperature range	(1,0,1) × ( 0,1,1)_12_	613.5	0.516
Monthly average of ambient temperature range within two days	(1,0,1) × (0,1,1)_12_	748.8	0.439
Monthly average of ambient temperature range within one week	(1,0,1) × (0,1,1)_12_	1299.5	0.134
Monthly average of daily mean dewpoint	(0,0,0) × (0,1,1)_12_	1630.5	0.317
Monthly average of daily mean amount of cloud	(0,0,0) × (0,1,1)_12_	3353.9	0.877
Adjusted monthly total number of sunshine hours	(0,0,0) × (0,1,1)_12_	4625.2	0.576
Adjusted monthly total rainfall	(0,0,0) × (0,1,1)_12_	5909.1	0.950
Adjusted monthly number of days with maximum ambient temperature ≥33 °C (defined by the Hong Kong Observatory as “very hot day”)	(0,0,1) × (0,1,1)_12_	1833.5	0.970
Adjusted monthly number of days with three consecutive days of maximum ambient temperature ≥33 °C	(0,0,1) × (0,1,1)_12_	1409.6	1.000
Adjusted monthly number of days with minimum ambient temperature ≥28 °C (defined by the Hong Kong Observatory as “hot night”)	(1,0,0) × (0,1,1)_12_	1923.8	0.994
Adjusted monthly number of days with three consecutive days with minimum ambient temperature ≥28 °C	(1,0,0) × (0,1,1)_12_	1420.9	0.983
Adjusted monthly number of days with Tropical Cyclone Warning Signals	(0,0,0) × (0,1,1)_12_	1844.7	0.959

**Table 3:** Transfer function models for the rate of older-adult suicide deaths from violent methods.

Meteorological Variables as Input	Best Transfer Function Model			SBC	*p*-Value of Ljung-Box Test (Lag 18)
	Lag	Numerator	Denominator		
Monthly average of daily maximum ambient temperature	0	1	1	−3077.2	0.463
Monthly average of daily mean ambient temperature	0	1	1	−3078.1	0.448
Monthly average of daily minimum ambient temperature	0	1	1	−3078.7	0.437
Monthly average of ambient temperature range within one week	2	0	1	−3040.6	0.227
Monthly average of daily mean dewpoint	0	0	0	−3076.4	0.307
Monthly average of daily mean amount of cloud	1	0	0	−3049.0	0.215
Adjusted monthly total number of sunshine hours	2	0	1	−3037.4	0.399
Adjusted monthly total rainfall	0	0	0	−3052.9	0.233
Adjusted monthly number of days with maximum ambient temperature ≥ 33 °C	1	1	1	−3028.9	0.094
Adjusted monthly number of days with Tropical Cyclone Warning Signals	0	1	1	−3048.4	0.388

**Table 4:** Transfer function models for the rate of older-adult suicide deaths from nonviolent methods.

Meteorological Variable as Input	Best Transfer Function Model			SBC	*p*-Value of Ljung-Box Test (Lag 18)
	Lag	Numerator	Denominator		
Monthly average of daily maximum ambient temperature	0	0	0	−3993.5	0.132
Monthly average of daily mean ambient temperature	0	0	0	−3993.2	0.133
Monthly average of daily minimum ambient temperature	0	0	0	−3992.9	0.135
Monthly average of ambient temperature range within one week	2	0	1	−3965.1	0.281
Monthly average of daily mean dewpoint	0	0	0	−3993.1	0.145
Monthly average of daily mean amount of cloud	2	0	0	−3978.7	0.337
Adjusted monthly total number of sunshine hours	1	1	0	−3980.3	0.320
Adjusted monthly total rainfall	0	0	1	−3980.0	0.113
Adjusted monthly number of days with maximum ambient temperature ≥ 33 °C	0	0	0	−3992.3	0.094
Adjusted monthly number of days with three consecutive days of maximum ambient temperature ≥ 33 °C	1	0	0	−3984.1	0.142
Adjusted monthly number of days with minimum ambient temperature ≥ 28 °C	0	0	0	−3992.6	0.082
Adjusted monthly number of days with Tropical Cyclone Warning Signals	0	0	1	−3982.8	0.223

## Appendix A

**Table A1:** Meteorological variables used in data analyses.

**I. Monthly Average**
i. daily maximum/mean/minimum ambient temperature;
ii. diurnal ambient temperature range (difference between the daily maximum and minimum ambient temperature);
iii. ambient temperature range over two days (the difference between the daily maximum and minimum ambient temperature of two consecutive days);
iv. ambient temperature range over one week (the difference between the daily maximum and minimum ambient temperature for seven consecutive days);
v. mean dewpoint;
vi. mean amount of cloud;
**II. Monthly Total (Adjusted for the Number of Days in Each Month) of:**
i. hours of sunshine;
ii. rainfall;
**III. Monthly (Adjusted for the Number of days in Each Month):**
i. number of days with a maximum ambient temperature of ≥ 33 °C (defined by Hong Kong Observatory as a “very hot day”);
ii. number of occasions there were three consecutive days with a maximum ambient temperature of ≥ 33 °C;
iii. number of days with a minimum ambient temperature of ≥ 28 °C (defined by Hong Kong Observatory as a “hot night”);
iv. number of occasions there were three consecutive days with a minimum ambient temperature of ≥ 28 °C;
v. number of days with a Tropical Cyclone Warning Signals (signal number 1 or above).

**Table A2:** Summary of key data used in the study.

Year	Annual Older-Adult Suicide Mortality Rate (per 100,000 Population) (per 100,000)		Average Daily Min. Ambient Temperature (°C)	Average Daily Max. Ambient Temperature (°C)	Total Number Of Days with Min. Ambient Temperature ≥28 °C	Total Number Of Days with Max. Ambient Temperature ≥33 °C
	Violent Methods	Nonviolent Methods				
1976	51.5	6.1	20.4	25.4	5	9
1977	42.9	5.4	21.1	26.4	14	17
1978	35.5	7.1	20.7	25.6	8	28
1979	41.5	5.6	21.0	25.8	10	14
1980	42.4	3.1	20.9	25.9	11	16
1981	26.4	3.8	21.2	25.5	10	5
1982	22.5	3.6	21.0	25.2	9	8
1983	31.0	2.1	21.1	25.4	30	13
1984	28.7	2.6	20.7	24.9	10	7
1985	34.0	3.4	20.8	25.0	2	2
1986	38.4	5.0	20.8	25.3	12	7
1987	38.6	2.3	21.6	25.7	20	9
1988	36.2	2.2	20.9	25.1	16	4
1989	33.4	3.8	21.1	25.3	17	15
1990	41.6	3.1	21.2	25.4	24	13
1991	37.2	2.6	21.6	25.9	19	12
1992	37.7	4.0	20.9	25.2	15	16
1993	35.0	3.7	21.2	25.5	24	6
1994	37.3	2.1	21.7	25.9	4	5
1995	35.1	2.7	20.9	25.2	15	7
1996	26.0	2.0	21.3	25.6	17	9
1997	32.3	1.6	21.5	25.5	12	5
1998	31.4	1.6	22.1	26.2	36	10
1999	26.2	2.4	21.8	26.2	17	6
2000	28.3	2.6	21.5	25.5	22	10
2001	27.2	2.7	21.8	25.8	16	9
2002	26.0	2.4	22.1	26.0	17	10
2003	32.7	4.9	21.9	25.7	20	14
2004	31.9	2.6	21.7	25.6	19	6
2005	28.9	1.6	21.4	25.4	26	12
2006	28.9	1.5	21.7	25.8	15	3
2007	27.1	1.7	21.7	26.4	23	25
2008	27.8	2.5	21.1	25.8	15	15
2009	27.0	1.8	21.6	26.3	28	30
2010	27.7	1.4	21.2	25.8	24	13
2011	24.0	1.9	21.0	25.8	21	19
2012	23.9	1.8	21.6	25.8	23	21
2013	22.3	1.7	21.4	25.9	10	17
2014	22.7	2.4	21.5	26.0	34	33
Trend	Decrease by 0.41 per year (*p* < 0.001)	Decrease by 0.08 per year (*p* < 0.001)	Increase by 0.02 °C per year (*p* < 0.001)	Increase by 0.01 °C per year (*p* = 0.010)	Increase by 0.35 days per year (*p* = 0.001)	Increase by 0.20 days per year (*p* = 0.054)

**Table A3:** Transfer function models for the rate of older-adult suicide deaths from violent methods (Sensitivity Analysis).

Meteorological Variables as Input	Best Transfer Function Model			SBC	*p*-Value of Ljung-Box Test (lag 18)
	Lag	Numerator	Denominator		
Monthly average of daily maximum ambient temperature ≤5th percentile (16.3 °C)	0	2	0	−3013.7	0.057
Monthly average of daily maximum ambient temperature ≤25th percentile (21.4 °C)	0	1	1	−3064.9	0.411
Monthly average of daily maximum ambient temperature ≥75th percentile (30.3 °C)	0	0	0	−3061.7	0.168
Monthly average of daily maximum ambient temperature ≥95th percentile (32.7 °C)	1	1	1	−3033.7	0.219
Monthly average of daily minimum ambient temperature ≤25th percentile (17.2 °C)	1	1	1	−3062.0	0.541
Monthly average of daily minimum ambient temperature ≥75th percentile (25.7 °C)	0	0	0	−3060.4	0.134

**Table A4:** Transfer function models for the rate of older-adult suicide deaths from nonviolent methods (Sensitivity Analysis).

Meteorological Variables as Input	Best Transfer Function Model			SBC	*p*-Value of Ljung-Box Test (lag 18)
	Lag	Numerator	Denominator		
Monthly average of daily maximum ambient temperature ≤5th percentile (16.3 °C)	0	0	0	−3991.7	0.126
Monthly average of daily maximum ambient temperature ≤25th percentile (21.4 °C)	0	0	0	−3993.0	0.141
Monthly average of daily maximum ambient temperature ≥75th percentile (30.3 °C)	0	0	0	−3992.3	0.119
Monthly average of daily maximum ambient temperature ≥95th percentile (32.7 °C)	0	0	0	−3994.4	0.087
Monthly average of daily minimum ambient temperature ≤5th percentile (12.1 °C)	0	0	0	−3991.3	0.120
Monthly average of daily minimum ambient temperature ≤25th percentile (17.2 °C)	0	0	0	−3992.6	0.153
Monthly average of daily minimum ambient temperature ≥75th percentile (25.7 °C)	0	0	0	−3993.9	0.101
Monthly average of daily minimum ambient temperature ≥95th percentile (27.9 °C)	0	0	0	−3992.9	0.089
